# Does Subclinical Hypothyroidism Affect Hospitalization Outcomes and Mortality in Congestive Cardiac Failure Patients?

**DOI:** 10.7759/cureus.2766

**Published:** 2018-06-08

**Authors:** Shanan Mahal, Sorabh Datta, Virendrasinh Ravat, Priya Patel, Bipin Saroha, Rikinkumar S Patel

**Affiliations:** 1 Department of Internal Medicine, Providence Hospital, Washington DC, USA; 2 Department of Infectious Disease, Clinical Infectious Disease Specialist, Las Vegas, USA; 3 Department of Psychiatry, Windsor University School of Medicine, Philadelphia, USA; 4 Internal Medicine, University of Chicago Medical Center, Chicago, USA; 5 Department of Psychiatry, Griffin Memorial Hospital (odmhsas), Norman, USA

**Keywords:** hospitalization, ccf, heart failure, outcomes, iatrogenic hypothyroidism, subclinical hypothyroidism

## Abstract

Objective

This study aimed to determine the differences in hospitalization outcomes among patients admitted for congestive cardiac failure (CCF) with underlying subclinical hypothyroidism (SCH).

Methods

This retrospective case-control study used data from the nationwide inpatient sample (NIS) for the years 2012–2014. We identified cases with CCF as the primary diagnosis and SCH as the secondary diagnosis using validated ICD-9-CM codes and controls with CCF only. The differences in hospitalization outcomes and hospital characteristics were quantified using the multinomial logistic regression model (adjusted odds ratio (aOR)).

Results

A total of 143,735 CCF patients were enrolled in this study, and 73,440 cases had IH. About 31.8% of SCH patients were hospitalized for more than four days (median) compared to 44.7% patients without SCH (P < .001). The median hospitalization charges per admission for CCF was $20,312. CCF patients with SCH had lower odds of longer hospitalization (aOR = .709, 95% CI .660-.762, P < .001) and higher hospitalization charges (aOR = .783, 95% CI .728-.841, P < .001) compared to CCF patients without SCH. CCF patients with SCH had two times higher odds of minor morbidity (aOR = 2.276; 95% CI 2.105-2.462; P < .001) but lower odds of major morbidity (aOR = .783; 95% CI .728-.841; P < .001). Inpatient mortality with SCH patients (2%) compared to 3.6% patients without SCH (P < .001). CCF patients with SCH had lower odds of in-hospital mortality (aOR = .547; 95% CI .496-.604; P < .001). CCF patients with SCH had higher odds of being seen in rural non-teaching hospitals (aOR = 1.696; 95% CI 1.572-1.831; P < .001). Also, CCF patients with SCH had the highest likelihood of presence in the western region of the United States (aOR = 149.924; 95% CI 110.497-203.419; P < .001) followed by the southern region (aOR = 31.431; 95% CI 26.066-37.900; P < .001).

Conclusions

Among CCF with SCH patients during hospitalization, we observed a variation in hospitalization outcomes, including inpatient length of stay and cost, morbidity, and in-hospital mortality. We found no significant increase in mortality and major morbidity in CCF patients with SCH. There were differences in the hospital characteristics between CCF patients with and without SCH. Thus, hospital bed size, location, and teaching status act as predictors for a co-diagnosis of SCH in CCF. Further research is needed to guide the development of clinical care models for targeting early diagnosis and treatment to determine whether thyroid hormone replacement would be beneficial for CCF patients with SCH and improve quality of care in these patients.

## Introduction

Subclinical hypothyroidism (SCH) is a condition in which an elevated serum thyrotropin level (TSH) is present in combination with the normal range of serum free T4 level. This condition occurs in 3% to 8% of the general population. The occurrence of subclinical hypothyroidism varies among people, with a higher incidence associated with increasing age, female sex, and iodine deficiency [1-2]. There is little evidence of the clinical importance and therapy (levothyroxine) for treating subclinical hypothyroidism with a mild elevation of serum TSH (<10 mIU/L). Treatment is generally recommended for persons 70 years of age or younger who have thyrotropin levels of at least 10 mIU per liter, although long-term benefits are still controversial [1-2].

Subclinical hypothyroidism falls under the category of primary hypothyroidism, which has an etiology that is very similar to overt hypothyroidism [3]. Most cases of SCH are caused by chronic lymphocytic thyroiditis (goitrous Hashimoto’s thyroiditis and atrophic thyroiditis). According to previous studies, an autoimmune disorder of the thyroid gland decreased thyroid hormone production in patients with acquired mild, subclinical hypothyroidism [3]. Other causes of primary hypothyroidism could be iatrogenic therapies like radioactive iodine treatment or external radiation therapy that destroy thyroid tissue [3].

Thyroid hormones have a wide range of effects on the human body, among which the cardiovascular system is one of the primary target organs of the thyroid hormone. The most prominent effect of hypothyroidism on patients is increased systemic vascular resistance and impaired systolic and diastolic cardiac function. This same study found that a slower rate of left ventricular relaxation might critically reduce ventricular filling during exercise and result in left ventricular systolic dysfunction [4]. Subclinical hypothyroidism is associated with an increased incidence of congestive heart failure (CHF) at TSH levels > 10 mIU/L. However, the effects of SCH in patients with pre-existing CHF have not been studied (Kannan L, Morley M, Brandimarto J, Cappola TP, and Cappola AR. Thyroid dysfunction in heart failure is associated with cardiovascular outcomes: the Penn Heart Failure Study. ENDO 2017. April 2, 2017). An older population with SCH and a high CV risk appears to be at an increased risk of incident heart failure and SCH. A recent cardiac magnetic resonance spectroscopic study demonstrated that early cardiac bio-energetic impairments in SCH patients are reversible with levothyroxine therapy [5].

In previous meta-analysis studies, the association between subclinical hypothyroidism and the risk of mortality did not differ among patients with and without underlying cardiac failure, whereas in a subsequent meta-analysis, pre-existing CHF was observed to modify the association between SCH and CHF events. Given the impact of low serum thyrotropin level on cardiac contractility, systemic vascular resistance, electrophysiologic irritability, and atherosclerosis, it has been speculated that subclinical hypothyroidism might predispose to death from cardiac causes. It is plausible that subpopulations with CHF may be predisposed to the cardiac morbidity and mortality associated with subclinical hypothyroidism, owing to underlying distortions in ventricular architecture, alterations in neurohormonal activation, and vascular tone [6].

To better understand the SCH role in CCF patients, this is a study to evaluate the impact of SCH on CCF patients during hospitalization. As there is a rising concern of SCH, we also determined the hospital characteristics that had a higher odds of the codiagnosis of SCH in CCF patients.

## Materials and methods

Data source

In the study mentioned below, a retrospective analysis was performed using the healthcare cost and utilization project (HCUP) nationwide inpatient sample (NIS) data [7]. The agency for healthcare research and quality (AHRQ) sponsors the HCUP databases that are specifically designed to determine and identify patterns in utilization and cost across the United States hospitals. The HCUP-NIS database is the largest inpatient database available in the United States, which represents a sample of non-federal United States community hospitals. The sample size available via the database further facilitates the recognition and analyses of rare conditions and special patient populations. The patients, physicians, hospitals, state, and hospital identifiers are de-identified, to protect the privacy of the individual. Also, there are many clinical and non-clinical hospitalization data elements recorded in the HCUP NIS database. The sample of non-clinical information includes the patient’s demographic data, hospital characteristics, and total inpatient charges. For example, the clinical-related information includes primary, and other, diagnoses, disposition status, and length of inpatient stay.

Variables of interest

Based on the International Classification of Diseases, 9th Edition, Clinical Modifications (ICD-9-CM) diagnosis codes, we identified the controls with a primary diagnosis of CCF at the time of admission. Correspondingly, based on the ICD-9-CM diagnosis codes, patients with a primary diagnosis of CCF and a secondary diagnosis of SCH at the time of admission were identified as the cases. In HCUP databases, more than 14,000 ICD-9-CM diagnosis codes had been mentioned. CCF, non-hypertensive type, was identified using diagnosis code 398.91, 428.0, 428.1, 428.20–428.23, 428.30–428.33, 428.40–428.43, or 428.9. Both iatrogenic hypothyroidism and acquired hypothyroidism are part of SCH [3], and, so, SCH was identified using ICD-9-CM diagnosis codes 244.3 (iatrogenic hypothyroidism) and 244.8 (other, specified, acquired hypothyroidism).

To measure the differences in hospitalization outcomes in CCF patients versus CCF with SCH patients, the outcome variables of interest included the severity of illness, which measures the loss of body functions, inpatient length of stay, inpatient total charges, disposition of the patient, and in-hospital mortality. In the NIS, we defined death as in-hospital mortality and, in this paper, it is reported as all-cause. We calculated the inpatient length of stay as the number of nights the patient remained in the hospital for a particular discharge and the inpatient stay was all-cause in this analysis. Inpatient total charges during hospitalization do not include professional fees and non-covered charges. If the source provided total inpatient charges with professional fees, the professional fees were removed from the charge during HCUP processing.

Case and control subject selection

Cases were selected from among all discharges in the NIS from 2012–2014 with a primary diagnosis of CCF, the secondary diagnosis of SCH and age > 18 years. Controls were selected from among all discharges in the NIS from 2012–2014 with a primary diagnosis of CCF and were matched with the cases for age, gender, and race.

Statistical analysis

In this study, exploratory data analysis was performed using cross-tabulation over the NIS database focusing on the patient with CCF and SCH and the use of descriptive statistics to summarize the results. Pearson’s chi-square test and independent sample T-test were used for categorical data and continuous data, respectively. Multilevel logistic regression with random effects for hospital variability was used to calculate adjusted Odds Ratio (aOR). We used the NIS database to obtain nationally representative inpatient data, and age, gender, and race were estimated. All significance tests were two-sided and a p-value < 0.05 was used as a reference to determine the statistical significance test result. A statistical analysis was conducted using Statistical Package for the Social Sciences (SPSS) version 23 (IBM, Armonk, NY, US) in this study [8]. Our study database does not contain any patient identification and authentication. Thus, we were not required to take Institution Review Board (IRB) permission for this study.

## Results

Sample characteristics

A total number of 143,735 CCF patients were enrolled in this case-control study from 2012-2014; 73,440 cases had subclinical hypothyroidism (SCH) and 70,295 controls did not have SCH. About half of the total CCF patients were above 80 years of age (50.3%, N= 72,270) with a very low proportion of patients below 60 years (11.6%, N= 16,760). The majority of the patients in this study were females (69.4%, N = 99,775) and Caucasians (78.8%, N = 110850). The demographic distribution of the sample population is mentioned in Table 1.

**Table 1:** Demographic distribution in CCF patients by SCH CCF: congestive cardiac failure; SCH: subclinical hypothyroidism

Variable	CCF		CCF + SCH		Overall Total	
	N	%	N	%	N	%
Age						
21-40 years	780	1.1	825	1.1	1605	1.1
41-60 years	7415	10.5	7740	10.5	15155	10.5
61-80 years	26745	38.0	27960	38.1	54705	38.1
> 80 years	35355	50.3	36915	50.3	72270	50.3
Gender						
Male	21540	30.6	22405	30.5	43945	30.6
Female	48755	69.4	51020	69.5	99775	69.4
Race						
White	55420	78.8	55430	78.8	110850	78.8
Black	6560	9.3	6560	9.3	13120	9.3
Hispanic	5215	7.4	5215	7.4	10430	7.4
Asian/Pacific Islander	1225	1.7	1225	1.7	2450	1.7
Native American	480	.7	485	.7	965	.7
Other	1395	2.0	1400	2.0	2795	2.0

Hospitalization outcomes differences per SCH

About 68,110 CCF patients with SCH (93.1%) were admitted based on an emergency condition or a non-elective basis, while 5,085 (6.9%) were admitted on an elective basis. CCF patients with SCH were two times more likely to be admitted on an emergency or elective basis compared to patients without SCH (OR = 2.407, 95% CI 2.208-2.625, P < .001).

The median length of inpatient stay per admission for CCF was four days and 31.8% of SCH patients (N = 23,370) were hospitalized for more than four days compared to 31,435 patients without SCH (44.7%) (P < .001). The median hospitalization charges per admission for CCF was $20,312. There was not a statistically significant difference between patients with SCH (49.4%, N = 36,300) and without SCH (49.3%, N = 34,645) having hospitalization charges more than $20,312 (P = .588). Thus, CCF patients with SCH had lower odds of longer hospitalization (OR = .709, 95% CI .660-.762, P < .001) and higher hospitalization charges (OR = .783, 95% CI .728-.841, P < .001) compared to patients without SCH. The differences in hospitalization outcomes and hospital characteristics are mentioned in Table 2.

**Table 2:** Hospital outcomes and characteristics distribution in CCF patients by SCH Significant P ≤ 0.05 at 95% confidence interval CCF: congestive cardiac failure; SCH: subclinical hypothyroidism

Variable	CCF		CCF + SCH		P
	N	%	N	%	
Admission type					
Non-elective	68430	97.4	68110	93.1	< .001
Elective	1835	2.6	5085	6.9	
Severity of illness/morbidity					
Minor loss of body function	5470	7.8	11535	15.7	< .001
Moderate loss of body function	28425	40.4	35855	48.8	
Major loss of body function	36400	51.8	26050	35.5	
Inpatient stay and cost					
Inpatient stay > 4 days (median)	31435	44.7	23370	31.8	< .001
Inpatient cost > $20312 (median)	34645	49.3	36300	49.4	.588
In-hospital mortality					
Inpatient deaths	2495	3.6	1480	2.0	< .001
Bed size of hospital					
Small	19360	27.5	13470	18.3	< .001
Medium	22620	32.2	18895	25.7	
Large	28315	40.3	41075	55.9	
Location/teachin status of Hospital					
Rural, non-teaching	6955	9.9	12035	16.4	< .001
Urban, non-teaching	23600	33.6	28790	39.2	
Urban, teaching	39740	56.5	32615	44.4	
Region of hospital					
Northeast	69380	98.7	9395	12.8	< .001
Midwest	575	.8	28810	39.2	
South	285	.4	19185	26.1	
West	55	.1	16050	21.9	

A lower proportion of CCF patients with SCH (35.5%, N = 26,050) had a major loss of body function or morbidity compared to 51.8% (N = 36,400) patients without SCH (P < .001). CCF patients with SCH had two times higher odds of minor morbidity (OR = 2.276; 95% CI 2.105-2.462; P < .001) but lower odds of major morbidity (OR = .783; 95% CI .728-.841; P < .001). Also, 1480 (2%) patients with SCH died during hospitalization compared to 2495 patients without SCH (3.6%) (P < .001). CCF patients with SCH had lower odds of in-hospital mortality (OR = .547; 95% CI .496-.604; P < .001). The association of adverse hospitalization outcomes in CCF patients with SCH are mentioned in Table 3.

**Table 3:** Association of adverse hospital outcomes in CCF with SCH patients Significant P ≤ 0.05 at 95% confidence interval, variables were Agency for Healthcare Research and Quality (AHRQ) comorbidity measures CCF: congestive cardiac failure; SCH: subclinical hypothyroidism

Variable	Odds Ratio	95% Confidence Interval		P
		Lower Bound	Upper Bound	
Moderate Morbidity	1.380	1.284	1.484	< .001
Major Morbidity	.783	.728	.841	< .001
Inpatient Stay > 4 days	.709	.660	.762	< .001
Inpatient Cost > $20312	1.013	.944	1.087	.715
In-hospital Mortality	.547	.496	.604	< .001

Hospital predictors for SCH co-diagnosis in CCF

More than half of the CCF patients with SCH (55.9%, N = 41,075) were seen in large-bed-size hospitals and urban teaching hospitals (44.4%, N = 32,615). Yet, CCF patients with SCH had higher odds of being seen in rural non-teaching hospitals (OR = 1.696; 95% CI 1.572-1.831; P < .001). The highest proportion of CCF patients with SCH were seen in hospitals located in the midwestern regions of the US (39.2%, N = 28,810), followed by the southern region (26.1%, N = 19,185), the western region (21.9%, N = 16,050), and then the northeastern region of the US (12.8%, N = 9,395). When compared with patients without SCH, CCF patients with SCH had the highest likelihood of presence in the western region of the US (OR = 149.924; 95% CI 110.497-203.419; P < .001) followed by the southern region (OR = 31.431; 95% CI 26.066-37.900; P < .001). Hospital predictors of SCH in CCF patients are mentioned in Table 4.

**Table 4:** Hospital predictors for SCH in CCF patients Significant P ≤ 0.05 at 95% confidence interval, variables were Agency for Healthcare Research and Quality (AHRQ) comorbidity measures CCF: congestive cardiac failure; SCH: subclinical hypothyroidism

Variable	Odds Ratio	95% Confidence Interval		P
		Lower Bound	Upper Bound	
Bed size of hospital				
Small	.574	.533	.619	< .001
Medium	.724	.673	.778	< .001
Large	1.258	1.171	1.351	< .001
Location/teaching type of hospital				
Rural, non-teaching	1.696	1.572	1.831	< .001
Urban, non-teaching	1.270	1.181	1.365	< .001
Urban, teaching	.831	.774	.892	< .001
Region of hospital				
Northeast	.056	.048	.065	< .001
Midwest	20.340	17,223	24.022	< .001
South	31.431	26.066	37.900	< .001
West	149.924	110.497	203.419	< .001

## Discussion

This analysis of population-based hospital data from patients admitted with CCF and CCF with SCH discloses the impact of SCH on hospitalization and related outcomes in CCF. More than half the patients were above the age of 80 years, which may be due to the increase in serum T4 and TSH levels, decreased triiodothyronine (T3) levels, and reverse triiodothyronine (rT3) levels with increasing age. In an earlier study on the thyroid hormone, it has been shown that with aging, the measurement of serum deiodinase levels in a range of healthy adults has demonstrated a significant inverse correlation of 3’,3’-diiodothyronine, 3’.5’-diiodothyronine, and 3,5-diiodothyronine levels. Also, it can be seen in elderly individuals due to the increasing incidence and prevalence of autoimmune thyroiditis that occurs with aging [9]. Studies also revealed more SCH in the female population according to the Whickham survey [10]. The prevalence of SCH was three times higher in women than in men. One possible reason for this difference is that the majority of cases of thyroid dysfunction are due to an autoimmune disease, which is more common in women. Therefore, it is necessary to routinely evaluate thyroid function among women during health examinations [10]. According to the American College of Physicians, treatment for subclinical thyroid dysfunction is controversial but suggests that screening to detect thyroid dysfunction may be indicated in women older than 50 years [11].

In an earlier study, the association between CCF patients with SCH shows that a TSH level of 7.0 mIU/L or greater was predictive of an incident and recurrent CCF events in those without and with a common diagnosis of CHF [12]. The controversy of the clinical benefit of levothyroxine in older patients in an SCH trial on the incidence of cardiovascular events or mortality indicated that treatment with levothyroxine in older persons with subclinical hypothyroidism provided no significant benefits [13].

In our study, a majority of the patients were admitted under emergency conditions and a higher proportion of CCF patients without SCH had a longer inpatient stay compared to patients with SCH, but equal proportions of SCH and non-SCH patients had hospitalization charges higher than the median. A CCF patient with SCH had higher odds of moderate morbidity compared to a patient without SCH. Yet, in-hospital mortality was also lower in CCF patients with SCH (2%) compared to patients without SCH (3.6%). Moreover, according to the other studies, it demonstrates that patients with SCH were associated with an increased risk for all-cause and cardiovascular mortality in adults [14]. Previous results showed that SCH may increase the risks of hypercholesterolemia and atherosclerosis, and a mildly altered thyroid status has been reported to be associated with an increased risk of mortality in patients with cardiac disease. This reported an increased risk of all-cause mortality in SCH patients with comorbid conditions [15]. There is a lot of debate over mortality among CCF patients with SCH, and our study did not appreciate any increase in the association with in-hospital mortality as CCF patients with SCH had lower odds of in-hospital mortality compared to patients without SCH.

A higher proportion of CCF patients with SCH were seen in large-size teaching hospitals in the urban region and the majority of these cases were present in the mid-western region (39.2%) of the United States. This study also found that CCF patients admitted in rural and non-teaching hospitals have higher odds of having SCH, and these patients in the western region of the United States have a 200 times higher likelihood of a co-diagnosis of SCH.

Limitations

NIS is an organizational database and the data used in these studies lacks the patient-level data needed to form specific clinical associations. Additionally, these types of retrospective case-control studies are always subject to a selection bias, which might be accentuated by the moderate sensitivity of diagnostic codes for CCF and SCH. In this study, we too could not account for the re-hospitalizations of patients, given the nature of the database, although they add to the total inpatient burden. Despite all these restraints, the NIS database provides an excellent population-based perspective on disease associations with systematic and temporal factors and present a rationale for more thorough studies. This study of the dataset is subject to at least a reporting bias, and all information is coded independently of the individual practitioner, making it a probably more reliable source.

## Conclusions

During the hospitalization of patients with CCF and SCH, we observed a variation in hospitalization outcomes, including inpatient length of stay and cost, morbidity, and in-hospital mortality. We found no significant increase in mortality and major morbidity in heart failure patients with subclinical hypothyroidism. There were differences in the hospital characteristics between CCF patients with and without SCH. Thus, hospital bed size, location, and teaching status act as predictors for a co-diagnosis of SCH in CCF. More attention needs to be paid to the elderly population and female sex, which is at a higher risk of SCH. Further research is needed to guide the development of clinical care models for targeting early diagnosis and treatment and to determine whether thyroid hormone replacement would be beneficial for CCF patients with SCH, to both further reduce mortality and morbidity and improve quality of care in these patients.
